# Modern Sociology

**Published:** 1899-05-27

**Authors:** 


### Modern Sociology.

### JUVENILE DELINQUENTS.

Of late the problem of how best to deal with juvenile offenders has aroused and is arousing much interest in the minds of thoughtful people. The system followed in England till within recent times, of committing mere children to gaol, there to mix with hardened criminals, stands self-condemned on the face of it, but it cannot be said that the attempts to treat young people in reformatory schools of various kinds are as successful as might be expected. The reason is not far to seek. The young inmates are treated in the mass; they feel themselves atoms in a huge mechanical system; they become "institutionalised," and when they leave the shelter to which they have been confided by the State they are entirely unfitted to battle with the world and to take their place therein as useful citizens.

In America, where social reforms are pushed forward with more vigour and speed than in our own land, tied and bound in so many ways as we are by bad old traditions, experience has proceeded on the same lines. The success which has attended an experiment tried in the State of New York, initiated by members of the Prison Association of New York, entitles it to serious consideration, as showing what may be done when the problem of juvenile depravity is faced by earnest workers in the cause of humanity.

The knowledge that children of tender years were locked up with older criminals, and that 40 per cent. of the youthful prisoners in the larger gaols had previously been in reformatories, first turned the attention of Mr. W. F. Round and his co-workers on the Prison Association to the needs of the children. A study of the prevailing system demonstrated that the reformatories were simply acting as "feeding schools" for the gaols—"devil's kindergartens," as Mr. Round called them in a recent lecture at the Sanitary Institute—almost, it might be said, adding to the mischief they existed to allay. Something had to be attempted to check the evil, and Mr. Round and his friends determined to strike along new lines and see what could be done. Mr. Frederick G. Burnham gave a large farm of 800 acres (it now numbers 1,000), an old Shaker settlement, for the purpose. Boys were gathered together from all parts of the country, "their only qualification being that they had shown such conduct of life as promised nothing but disaster, and such qualities as made it hopeful that they might be saved if rightly treated." The difficulties of organising such a scheme as the founders had in view were necessarily great, that of finding the men qualified by character and training to undertake the superintendence being one of the most formidable. Then, with the example of Dr. Wichern and the "Rauhe Haus" before them, the trustees published a notice of their wants, offering "no pay, hard work, and long hours" to young men ready to devote themselves to the service of humanity. Twenty-nine responses came, and on January 1st, 1890, the Brotherhood or Order of St. Christopher was founded, the name being chosen because the legend of St. Christopher "seemed to fit the idea of the work to be done." And so the Burnham Industrial Farm was established at Canaan Four Corners, Columbia co., New York, nearly ten years ago, and worked by these devoted men. The "Brothers" are "unsectarian Christians," bound together by the simplest rules, and a promise to devote three and a half years to their training.

It was designed to receive boys between the ages of seven and sixteen, whose habits and surroundings would inevitably tend to lives not only of uselessness to society, but of positive evil and crime. The vital principle upon which the whole scheme rested was that the boys should not "be brought merely under the influence of cast-iron discipline, the accompaniments of which would naturally consist of high walls and barred gates, but to remove them from pernicious surroundings and bring them under influences closely allied to those of a home circle, and so achieve a lasting moral improvement." The conditions at the Burnham Farm, in fact, approximate as nearly as possible to those of ordinary family and social life.

Acting on this idea, every boy who enters the farm is expected to work, and is paid for his work, on a small scale, from the moment of his arrival. Only the commonest necessaries of food and clothing are provided, additions and luxuries having to be bought by the boys with the money they earn. The use of the library, a share in the games, membership of club and guild depend upon each boy himself, a deposit from their wages being a necessary qualification for these various privileges. The wages are raised as each boy improves in his trade or calling, while those who will not work are treated as "paupers," their companions being taxed for their support. By this plan it may be imagined that there are not many "paupers," for the boys whose pockets suffer by their idleness see well to that! The farm is a self-contained little village, with its general store (here the boys can buy their luxuries), carpenters' shop, printing office, shoe shop, dairy, farm, garden, all worked by the boys under efficient management; and there is also a fire department, and a police department, whilst military training under an expert drill master forms part of the system. Cases of breach of discipline are tried by the boys, and the penalty sometimes determined also by them. There are no walls or locked doors at the Burnham Industrial Farm. Kindness and moral influence act instead of bolts and bars. To quote from the annual report for last year: "This is this peculiar work of the Berkshire Farm—not to punish, not strictly speaking to compel reform, but to change the boy's thoughts, wishes, hopes, and life from evil to good, before the set of the current has become too strong; to take him from his own down-pulling associations, and put him where the air, land, ideas, talk, hopes, and life are entirely different, and where a healthy, happy, manly, Christlike undertone will draw him as he has never felt it before."

Of course, all this means expenditure. Yet if it be expenditure which will turn boys into good citizens, and so make it impossible for them in after years to become chargeable to the State again and again through lapses into crime, is not the money well spent? And the Burnham Farm can point to a triumphant record of success in 90 per cent. of the cases committed to its fostering care, a percentage which would make almost any cost justifiable.

